# Special Issue: “Fungi: What Have We Learned from Omics?”

**DOI:** 10.3390/jof8111145

**Published:** 2022-10-28

**Authors:** Ana Cristina Esteves

**Affiliations:** CESAM and Departament of Biology, Campus Universitário de Santiago, 3810-193 Aveiro, Portugal; acesteves@ua.pt

Fungi are vast in terms of diversity, ecological roles, habitats they occupy, physiology, metabolism, and in many other characteristics. Most recent estimations predict that there are approximately 2.2 to 3.8 million species of fungi. With 120,000 currently known species, it appears that (at best) just 8%, and at the very worst just 3%, have been identified so far [1]. The plethora of metabolic processes occurring in fungi can only be untangled using diverse technologies and, often, multidisciplinary approaches. Fungi show an enormous interplay of proteins, metabolites, and lipids that interact with each other in a still-mysterious dance.

Proteomics, the first omics technology, (although only coined in 1995) began in 1975 with the work of O’Farrell [2], Klose [3], and Scheele [4] and can be considered the “mother” of omics. However, it was only with the advent of mass spectrometry that it truly took off. Genomics followed shortly after, when in 1977, Sanger developed the DNA Sanger sequencing technique [5], for which it won the Nobel prize in Chemistry, in 1980. Nowadays, a panoply of omics-related techniques exist: transcriptomics, interactomics, proteogenomics, lipidomics, even data analysis and bioinformatics that allow the intrinsic metabolic networks of complex organisms such as fungi to be characterized.

This Special Issue “Fungi: what have we learned from omics?” aimed to reveal what and how omics have contributed to unravelling fungal complexity. This e-book comprises a collection of 10 articles, covering research on fungi from different habitats, from plant-colonizing to marine fungi, fungi with different lifestyles (pathogenic and endophytic), as well as on the effect of abiotic factors on fungi. Most contributions to the Special Issue present the results of original research, but the e-book also includes one review article. Most studies addressed questions related to the interaction of phytopathogenic fungi with their hosts. Nazar Pour [6] and Kiselev [7] focused on the secretome of phytopathogenic fungi to unravel the infection mechanisms of these organisms. Nazar Pour identified secreted proteins of the phytopathogen *Neofusicoccum parvum* that are related to the colonization and infection of the host, simultaneously showing that *N. parvum* has the molecular apparatus to colonize and actively feed on its host living cells and induce necrosis, suggesting that *N. parvum* has an hemibiotrophic lifestyle. Kiselev, on the other hand, focused on the predicted secretome, sequencing the genome of strains of *A. euteiches*, with different geographical origins. These authors were able to detect minor differences that could be related to the pathogen origin and host specificity. Batool [8] and Wu [9] investigated the phytopathogen *Magnaporthe oryzae*. *Magnaporthe oryzae* is an ascomycete pathogen that causes blast disease in plants, especially economically relevant cereal crops. Batool used RNA Seq analysis to address the molecular mechanisms of the phytopathogen *M. oryzae* and its relation to osmotic stress and cold temperatures. The Serine threonine protein kinase AGC/AKT was found to be essential for fungal-infection-related morphogenesis as it is essential for the overwintering of *M. oryzae*. Wu sequenced the genomes of more than one hundred strains isolated from different plant hosts. Using a comparative genomics approach, the authors suggested that rice blast fungi adapted to rice by gene loss and rapid evolution of specific *loci*.

Fungi may also establish mutualistic relationships with plants. This is the case with *Diaporthe fraxini* which is an endophytic of the herb *Orthosiphon stamineus*. Tan [10] used LC-HRMS metabolomics to access the production of bioactive and antioxidant compounds by *D. fraxini* in supplemented media. *D. fraxinimetabolome* was found to be highly dependent on the culture media, representing a sustainable production method of natural products. The production of bioactive compounds was also addressed by Gonçalves [11].

Using an untargeted metabolomics approach (UPLC–QToF–MS/MS) and genome sequencing, the authors showed that *Emericellopsis cladophorae* produces antifungal (e.g., 9,12,13-Trihydroxyoctadec-10-enoic acid, hymeglusin), antibacterial (e.g., NovobiocinA), anticancer (e.g., daunomycinone, isoreserpin, flavopiridol), and anti-inflammatory (e.g., 2′-O-Galloylhyperin) metabolites, as well as many non-identified compounds whose expression is modulated by the presence of sea salts. Biotic factors such as osmotic pressure or oxygen concentration modulate the phenotype of fungi. Fungi of the order Mucorales are dimorphic. These organisms change between a coenocytic mycelium and a multipolar yeast-like form depending on oxygen concentration, which seems to be related to its pathogenicity. RNA from Mucor lusitanicus aerobic and anaerobic cultures were screened for gene expression (RNA seq by Illumina MiSeq) by Homa [12]. Although most transcripts could not be identified, the changes in the gene expression, when the culture has been transferred from aerobic to anaerobic conditions, were mainly related to an increase in the expression of carbohydrate-metabolism-related genes. Interestingly, only a small number of transcripts with putative regulatory roles in dimorphism were identified as both up- and down-regulated.

Edible fungi at times pose a difficulty in what concerns their postharvest preservation. Low-temperature storage is the traditional storage method used for most edible fungi. However, *Volvariella volvacea* undergoes autolysis at low temperatures, with a senescence mechanism that is unclear. Using iTRAQ-based proteomic quantitative analysis and interactomics, Zha [13] showed that, during storage, *V. volcacea* fruiting bodies decrease their gene regulation and expression, while H_2_O_2_ scavenging decreases and lipid peroxidation increases, contributing to maturation.

The metabolic alterations induced by starvation on Saccharomyces cerevisae was reviewed by Nasaruddin [14]. Reviewed literature included targeted and untargeted MS-based metabolomics and addressed nitrogen, glucose, and phosphate starvation. The authors identified common pathways to all the mutants studied, e.g., depletion of RNAs, and at the same time highlighted some limitations of the studies and some future directions to research on autophagy.

This Special Issue and the resulting e-book represent an important contribution to the current state of the art in the field of fungi omics. We hope that it will also help to identify relevant gaps in current knowledge and thus direct and promote new research in this exciting field.

## References

[1] Hawksworth D.L., Lücking R. (2017). Fungal diversity revisited: 2.2 to 3.8 million species. Microbiol. Spectr..

[2] O’Farrell P.H. (1975). High resolution two-dimensional electrophoresis of proteins. J. Biol. Chem..

[3] Klose J. (1975). Protein mapping by combined isoelectric focusing and electrophoresis of mouse tissues. A novel approach to testing for induced point mutations in mammals. Humangenetik.

[4] Scheele G.A. (1975). Two-dimensional gel analysis of soluble proteins. Characterization of guinea pig exocrine pancreatic proteins. J. Biol. Chem..

[5] Sanger F., Coulson A.R. (1975). A rapid method for determining sequences in DNA by primed synthesis with DNA polymerase. J. Mol. Biol..

[6] Nazar Pour F., Pedrosa B., Oliveira M., Fidalgo C., Devreese B., Driessche G.V., Félix C., Rosa N., Alves A., Duarte A.S. (2022). Unveiling the secretome of the fungal plant pathogen *Neofusicoccum parvum* induced by in vitro host mimicry. J. Fungi.

[7] Kiselev A., San Clemente H., Camborde L., Dumas B., Gaulin E. (2022). A comprehensive assessment of the secretome responsible for host adaptation of the legume root pathogen *Aphanomyces euteiches*. J. Fungi.

[8] Batool W., Liu C., Fan X., Zhang P., Hu Y., Wei Y., Zhang S.-H. (2022). AGC/AKT Protein kinase SCH9 is critical to pathogenic development and overwintering survival in *Magnaporthe oryzae*. J. Fungi.

[9] Wu Q., Wang Y., Liu L.-N., Shi K., Li C.-Y. (2022). Comparative genomics and gene pool analysis reveal the decrease of genome diversity and gene number in rice blast fungi by stable adaption with rice. J. Fungi.

[10] Tan W.-N., Nagarajan K., Lim V., Azizi J., Khaw K.-Y., Tong W.-Y., Leong C.-R., Chear N.J.-Y. (2022). Metabolomics analysis and antioxidant potential of endophytic Diaporthe fraxini ED2 grown in different culture media. J. Fungi.

[11] Gonçalves M.F.M., Hilário S., Van de Peer Y., Esteves A.C., Alves A. (2022). Genomic and metabolomic analyses of the marine fungus Emericellopsis cladophorae: Insights into saltwater adaptability mechanisms and itsbiosynthetic potential. J. Fungi.

[12] Homa M., Ibragimova S., Szebenyi C., Nagy G., Zsindely N., Bodai L., Vágvölgyi C., Nagy G., Papp T. (2022). Differential gene expression of mucor lusitanicus under aerobic and anaerobic conditions. J. Fungi.

[13] Zha L., Chen M., Guo Q., Tong Z., Li Z., Yu C., Yang H., Zhao Y. (2022). Comparative proteomics study on the postharvest senescence of *Volvariella volvacea*. J. Fungi.

[14] Nasaruddin M.L., Tajul Arifin K. (2021). Application of metabolomics in the study of starvation-induced autophagy in *Saccharomyces cerevisiae*: A scoping review. J. Fungi.

